# Supplementary material for: Clinical Workflow of Cone Beam Computer Tomography-Based Daily Online Adaptive Radiotherapy with Offline Magnetic Resonance Guidance: The Modular Adaptive Radiotherapy System (MARS)
Source: Cancers (Basel). 2024 Mar 19;16(6):1210. doi: 10.3390/cancers16061210 (PMC10969008; doi:10.3390/cancers16061210)
Supplement: Supplementary file 1 [file cancers-16-01210-s001.zip › cancers-2874051-supplementary.pdf]

Supplementary Data:

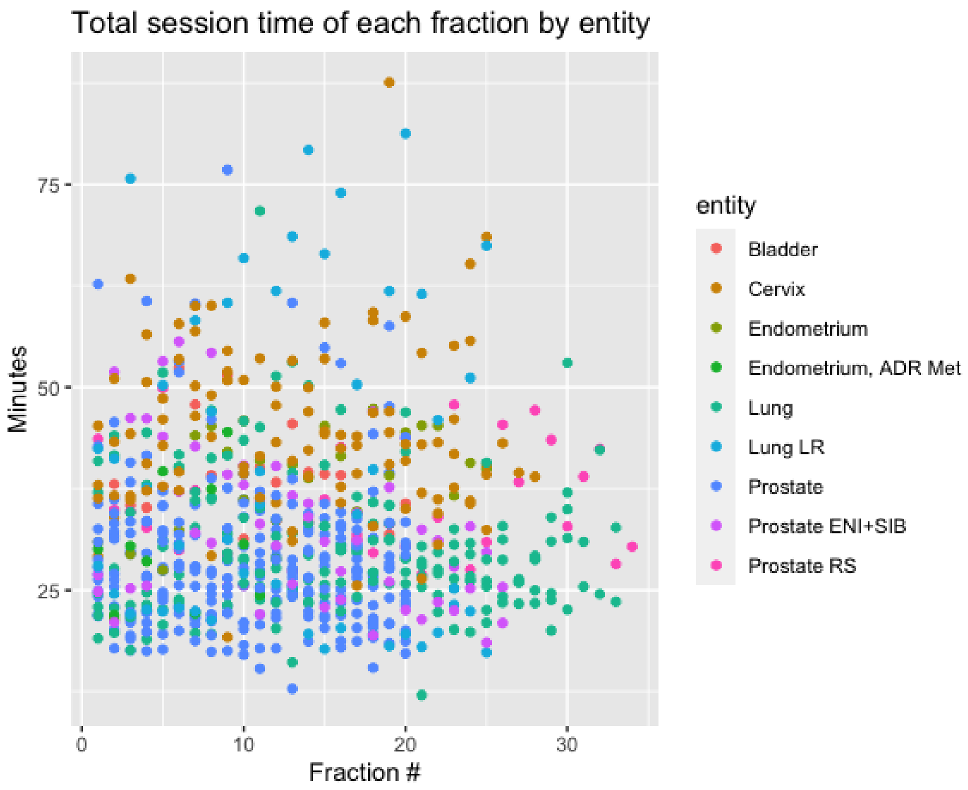

Figure S1. Total session time of Ethos oART adaptive sessions (n = 720). Entities refer to the different treatment concepts listed in Table 2. Abbreviations: ADR Met: adrenal gland metastasis, ENI + SIB: elective nodal irradiation + simultaneous integrated boost, LR: local recurrence, RS: rectal surgery

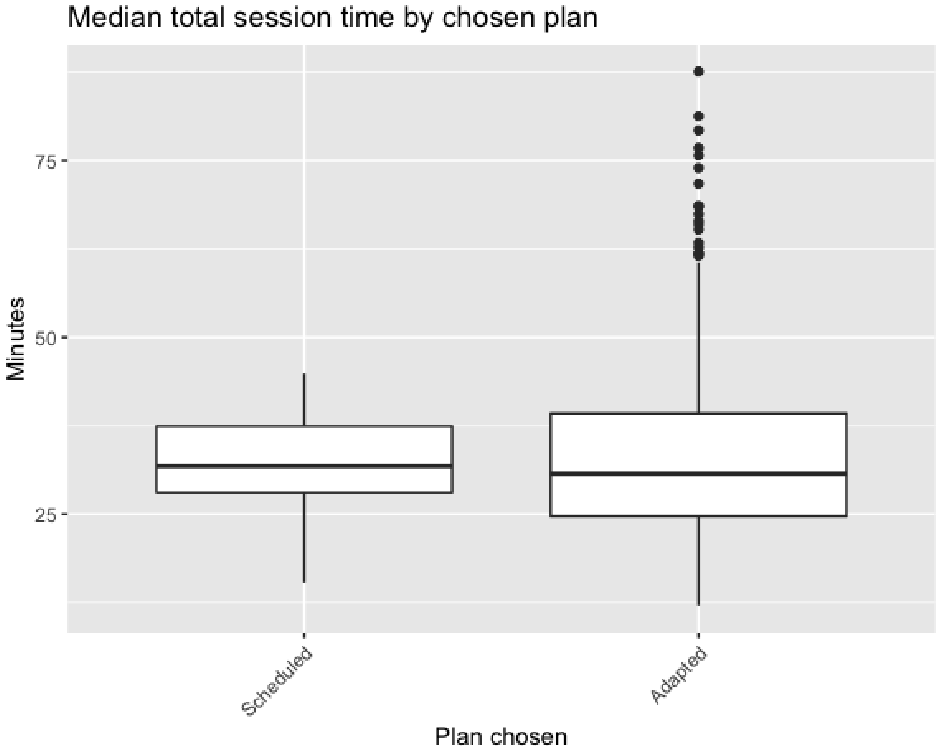

Figure S2. Median total session time by chosen plan. Numbers of fractions were scheduled (n = 17) and adapted (n = 720). Outliers >120 minutes (n = 1) are not shown but were included in the analysis.
